# Herbal synergy enhances growth performance, antioxidant status, immunity, and lymphoid tissue architecture in pigeons

**DOI:** 10.1038/s41598-025-26977-z

**Published:** 2025-12-03

**Authors:** Rasha I. M. Hassan, Hala Y. Amer, Walaa M. S. Gomaa, Ramadan D. EL-Shoukary, Abeer M. Hassan, Asmaa A. Rayan, Fatma El-Zahraa A. Mustafa

**Affiliations:** 1 Department of Nutrition and Clinical Nutrition, Faculty of Veterinary Medicine, Assiut University, Assiut, 71515 Egypt; 2 Department of Animal Nutrition and Clinical Nutrition, Faculty of Veterinary Medicine, New Valley University, El-Kharga, 72511 Egypt; 3 Department of Animal Hygiene, Faculty of Veterinary Medicine, New Valley University, El-Kharga, 72511 Egypt; 4 Department of Food Hygiene, Safety and Technology, Faculty of Veterinary Medicine, Assiut University, Assiut, 71526 Egypt; 5 Department of Microbiology and Immunology, Faculty of Veterinary Medicine, Assiut University, Assiut, 71526 Egypt; 6 Department of Cell and Tissues, Faculty of Veterinary Medicine, Assiut University, Assiut, 71515 Egypt

**Keywords:** Biochemistry, Immunology, Physiology, Zoology

## Abstract

This study evaluated the effects of a herbal mixture (HM) composed of black seeds, dill, sage, and coriander on pigeon squabs and their parents. Using a randomized design, 54 squabs were divided into three groups. All groups were fed a basal diet, and HM was added to groups 2 and 3 at 1% and 2%, respectively. To receive crop milk, squabs were caged with their parents. Squab performance and the self-maintenance behaviors (sleeping and preening) of their parents were positively affected by HM supplementation (*P* = 0.001). Blood metabolites, including cholesterol, triglycerides, and low-density lipoprotein cholesterol (LDL-C), were significantly decreased (*P* = 0.001). Antioxidation biomarkers glutathione peroxidase (GSH-Px) and superoxide dismutase (SOD) were positively stimulated. The meat’s chemical composition, including fat, protein, amino acids, and moisture, was influenced by HM (*P* = 0.004). Histomorphometrical investigation of the bursa and spleen showed the significant effects of HM on the follicle area, medulla, white pulp area, and their S100-positive cells. Overall, adding HM, especially at a 1% concentration, was more cost-effective and improved pigeon parents’ behavior, squabs’ performance, immunity, antioxidant status, meat quality, and bursal and spleen histology and immunohistochemistry.

## Introduction

Pigeon domestication began approximately 5,000 years ago; however, pigeons have lived alongside humans for thousands of years, serving as message carriers or sources of food^1^. In ancient civilizations, pigeons played a significant role in culture and religious beliefs^2^. Squab (young pigeon) meat is better than other types of poultry because it is rich in polyunsaturated fatty acids, essential amino acids, and minerals^3^, and it features higher protein and lower fat contents^4^. Squabs rely on receiving crop milk from their parents for development and growth^5^. Thus, imbalanced or insufficient nutrients in the diet of parent pigeons not only affect them but also affect their squabs, as it may lead to poor feed conversion, slow growth, increased susceptibility to diseases, and consequently higher mortality rates^3^. Therefore, feeding pigeon parents a balanced diet that includes all the nutrients they need to sustain their squab production is crucial^6^.

Natural additives, such as herbs, are essential to the development of the poultry industry because they can enhance feed conversion, weight gain, immunity, production, and overall bird health^7^. Their immunomodulatory, antimicrobial, antioxidant, anti-inflammatory, growth-promoting, and digestive system-stimulating effects have been investigated^8^. Furthermore, scientists have found that growth-promoting herbs are more economical, available, and safer than antibiotics, with no risk of contamination with egg or meat residues^9^.

One of the most extensively grown herbs each year is black seed (Nigella sativa L.)^10^. It is regarded as one of the most effective natural growth promoters used in the poultry industry^11^ due to its bioactive polyphenolics, including quercetin, protocatechuic acid, rutin, kaempferol, epicatechin, and ellagic acid^12^. These compounds contribute to the growth-promoting, immunomodulatory, anti-inflammatory, antioxidant, antiparasitic, and antibacterial properties of black seed^13,14^.

Recent research has emphasized the effectiveness of another important herb, dill seeds (Anethum graveolens)^15^. Dill, as a plant, has a high content of phenols, minerals, and vitamins^16^. Ali et al.^17^ investigated essential oils from herbs such as dill and coriander, reported improvements in overall health in broilers. Also, the beneficial effects of dill and coriander were linked to their anti-inflammatory and antioxidant properties. The primary phytochemicals responsible for dill’s anti-inflammatory and antioxidant effects are carvone and limonene^18^.

Sage (Salvia officinalis) is another medicinal herb known for its high essential oil content, which can stimulate blood metabolites, enhance digestion and gut health, inhibit inflammation and microbial activity, and induce hypoglycemic and hypolipidemic effects^19,20^. Rasouli et al.^21^ noted that sage plants can serve as a replacement for traditional antibiotics in specific applications. Todorova et al.^22^ reported that adding dried sage to the diet of laying hens enhanced weight gain and egg quality.

Another herb of interest used as a flavoring is coriander (Coriandrum sativum L.)^23^. In addition to its use as a medicinal herb due to its high effectiveness in preventing microbial infections, inflammation, and oxidation^24^. Coriander enhances nutrient digestibility and immune response in several poultry studies^25–27^.

To our knowledge, the herbal mixture of black seed, dill, sage, and coriander has not been studied in pigeon nutrition. Additionally, a few studies investigated the effect of herbal synergy on squabs via parental feeding, in which food is transferred from breeders to squabs via the crop. Moreover, research assessing the immune organs in pigeons using histological and immunohistochemical methods under herbal supplementation is very limited. Therefore, we hypothesize that adding HM of black seed, dill, sage, and coriander in a 1:1:1:1 ratio to the diet of pigeon parents might influence their behavior and the performance, immunity, antioxidant status, meat quality, and histology and immunohistochemistry of the bursa and spleen of their squabs.

The present study was designed to determine the impact of dietary supplementation with a HM of black seeds, dill, sage, and coriander on performance, carcass traits, blood metabolites, antioxidant levels, immunity, meat quality, histology, and immunohistochemistry of pigeon squabs, and the behavior of their parents.

## Materials and methods

The procedure of our experiment was carried out following the ethical standards and guidelines of the Faculty of Veterinary Medicine Ethics Committee, Assiut University, Egypt (06/2025/0348).

### Herbal mixture, birds, diets, and experimental design

The HM applied in this experiment, which consisted of black seeds, dill seeds, sage powder, and coriander seeds in a 1:1:1:1 ratio, was obtained from a commercial supplier (Imtenan Company, Assiut, Egypt).

Fifty-four one-day-old squabs were allocated into three groups using a randomized design. Each treatment group included 3 replicates of 6 squabs each. The basal diet was provided to all groups, while the HM was added to groups 2 and 3 at concentrations of 1% and 2%, respectively. The squabs were distributed into cages, each containing two squabs and two parent pigeons (one male and one female). The pigeons (Egyptian Baladi pigeons) were obtained from a commercial pigeon farm (Cairo, Egypt). All groups were provided with the basal mash diet, while the HM was added to groups 2 and 3 at concentrations of 1% and 2%, respectively. Throughout the 28-day experimental period, each pair of pigeon breeders, together with their squabs, was placed in separate wire cages (50 cm length × 50 cm depth × 60 cm height). Nests, feed, and water tanks were supplied in all cages, so parent pigeons had unrestricted access to food and water. Meanwhile, squabs relied on crop milk via parental beak-to-beak feeding. The pigeons received 18 h of light per day. The temperature was maintained at 22–25 °C, with relative humidity between 55 and 65% throughout the trial period. The birds’ routine veterinary care and thermoregulatory requirements were administered throughout the 28-day experimental period. The basal diet’s ingredients, formulated according to Omar et al.^28^ and Amer et al.^29^, are shown in Table 1.

**Table 1:** Ingredients and chemical composition of the basal diet.

Ingredients, g/kg as fed	Basal diet*
Yellow corn^1^	754.2
Soybean meal^2^, 45% CP	210.0
Dicalcium phosphate	16.00
Limestone, ground	12.30
Methionine	0.40
lysine	0.50
Salt	3.00
Premix^3^	3.00
Chemical composition (g/kg, as fed)	
Crude protein	160.87
Crude fiber	30.40
Ether extract	42.00
Calcium	12.00
Available phosphorus	4.00
Lysine	8.00
Methionine	3.00
Metabolizable energy^4^, kcal/kg	2994.87

* Basal diet was fed to the three groups of pigeons, with the herbal mixture added at 1% and 2% to the 2nd and 3rd groups, respectively. The herbal mix consisted of black seeds, dill seeds, sage powder, and coriander seeds at 1:1:1:1.

^1^ Composition of yellow corn (as fed basis): 88.45% DM, 8.80% CP, 2.22% CF, 4.68% EE, and 3350 kcal/kg diet ME.

^2^Composition of SBM (as fed basis): 88.96% DM, 45% CP, 6.55% CF, 1.00% EE, and 2230 kcal/kg diet ME.

^3^ Supplied per kilogram of diet: Vit. A, 6,250,000 IU; Vit.D3, 2,500,000 IU; Vit. E, 25,000 mg; Vit.k3, 1750 mg; Vit.B1, 500 mg; Vit.B2, 2750 mg; Vit.B6, 1250 mg; Vit. B12, 10 mg; Nicotinic acid 20,000 mg; calcium pantothenate, 500 mg; Folic acid 500 mg; Biotin 50 mg; Iron 22 g; Copper 2.5 g; Zinc 37.5 g; Manganese 31 g; Iodine 650 mg; Selenium 113 mg; cobalt 50 mg.

^4^ Metabolizable energy was calculated according to NRC (1994).

### Growth performance and carcass characteristics

Feed intake was recorded weekly for squabs and breeding pigeons. The body weights of the parent pigeons and squabs were taken on the following days: 1, 14, and 28. For each replicate, the following parameters were calculated at the end of the experiment: average daily feed intake, parent pigeons’ weight loss, squabs’ average daily gain (ADG), and nest feed conversion ratio (NFCR) (NFCR = total feed intake per nest of pigeons/total weight gain of breeding pigeon and squabs) according to Chen et al.^30^.

Following the approved protocol, squabs (three / treatment) were euthanized at the study end via jugular bleeding (2 min). Following evisceration, the weights of the dressed carcass, abdominal fat, and internal organs (liver, gizzard, proventriculus, pancreas, heart, spleen, bursa of Fabricius, and thymus) were recorded and expressed as a proportion of the body weight (BW):

### Behavior assessment

Behavioral data for both parents were collected via direct observation using the scan sampling technique^31^, with twice-daily sessions (8:00–9:00 AM and 2:00–3:00 PM, with 20 min/treatment/session) over two consecutive days each week. The behavioral satiety sequence (feeding, drinking, preening, and sleeping), as described by Spudeit et al.^32^, was observed.

### Serum biochemical indices, antioxidant biomarkers, and Immunoglobulins

At the end of the experiment, jugular venipuncture was used to collect blood samples from 3 pigeon squabs per treatment. Serum was separated from the collected blood samples using a centrifuge (3000 x g max. RCF, 100–4400 rpm sp., Millipore Sigma, USA) at 3000 rpm for 10 min, then stored at -20 °C for further examination.

Triglycerides, total cholesterol (T. cholesterol), LDL-C, high-density lipoprotein cholesterol (HDL-C), total protein, and albumin were analyzed according to standardized protocols using Chemistry Analyzer (BT 1500 Biotechnica Instruments, Via Licenza, Roma) with commercial kits (Egyptian Company for Biotechnology, Cairo, Egypt).

Antioxidant biomarkers, GSH-Px and SOD, were quantified with commercially available colorimetric kits (Egyptian Company for Biotechnology, Cairo, Egypt) using a spectrophotometer (Unico UV, 2000; Spectra Lab Scientific Inc., VA, USA).

Immunoglobulins G (IgG) and immunoglobulin M (IgM) were determined using the MAGLUMI 2000 analyzer (Snibe Diagnostics, Shenzhen, China).

### Meat quality assessment

Thigh and breast tissue from 3 squabs/group were cut into small pieces and thoroughly mixed in a mortar for subsequent meat chemical analysis, sensory examination, and measurement of quality parameters.

The pH levels of meat tissues were assessed with a pH meter (JENWAY 3505 pH meter, England), in line with the procedure outlined by Leroi et al.^33^. Color and odor were rated using a 9-point hedonic scale^34^. Drip loss was assessed utilizing methods established by Nkukwana et al.^35^ and Mahmoud et al.^36^. A technique from Honikel & Kim^37^ and Rahman & Kim^38^ was employed to gauge cooking loss. Water holding capacity (WHC) was measured using a modified approach^39,40^, which focused on fluid loss under muscle pressure.

Based on AOAC^41^ methods, a proximate analysis was conducted on squab samples to assess moisture, crude protein, fat, and ash content. The amino acid determination in the squab meat samples was performed as described by Li et al.^42^ using an amino acid analyzer (L-8900, Hitachi, Japan).

The meat oxidation analysis, including peroxide value and thiobarbituric acid reactive substances (TBARS), was performed^43–45^.

### Histology and immunohistochemistry

At the end of the experiment, tissue samples from the bursa of Fabricius and the spleen of squabs were collected and fixed in 10% formalin. The fixed samples were dehydrated in alcohol, cleared in methyl benzoate, embedded in paraffin wax, and sectioned at 4–5 μm. Sections were stained with Harris hematoxylin and eosin^46–48^. The following measurements were taken using ImageJ software:

- Bursa: area of follicle/um^2^, area of follicle medulla/um^2^, area of follicle cortex/um^2^, number of follicles/500 um, and S100 positive cell count.
- Spleen: white pulp/um² area and S100 positive cell count.

Immunohistochemistry was carried out on the paraffin-embedded sections using antibodies S100 (1:100; Catalog No.: A21069) and counterstained with Mayer’s hematoxylin^49–51^.

### Economic efficiency

The indices of economic evaluation included total feed costs, total revenue from bird sales, net revenue (returns minus costs), economic efficiency (EE, net returns divided by total costs multiplied by 100), and relative EE (the group’s efficiency compared to the control group, also multiplied by 100), based on the methodologies of Hassan & El Shoukary^52^ and Abdel-Raheem et al.^53^.

### Statistical analysis

The results were analyzed using one-way ANOVA with SPSS 16.0 software (SPSS Inc., Chicago, IL). Pooled SEMs were assessed using Duncan’s multiple-range test, with P values < 0.05 considered statistically significant.

## Results

### Growth performance and carcass characteristics

Table 2 shows the effect of the HM on the growth performance and carcass characteristics of pigeon squabs. BW remained unaffected by dietary supplementation with 1% or 2% of the HM on day 14. However, by the end of the experiment (day 28), both treatment groups exhibited a significant increase in BW compared to the control group. ADG was significantly increased (*P* < 0.05) with HM treatments.

**Table 2:** Effect of herbal mixture on the growth performance and carcass characteristics of pigeon squabs.

Item	Treatment*				
	T1	T2	T3up	SEM	P-value
Squabs					
BW(g), 1 d	20.70	20.10	20.60	0.73	0.827
BW (g), 14 d	303.00	300.30	297.20	11.15	0.935
BW (g), 28 d	367.20^b^	426.20^a^	416.80^a^	8.53	< 0.001
ADG of squabs (g/d)					
1-14 d	20.16	20.01	19.76	0.77	0.930
14-28 d	4.58^b^	8.99^a^	8.54^a^	0.69	< 0.001
1-28 d	12.37^b^	14.50^a^	14.15^a^	0.29	< 0.001
Pair of breeding pigeon					
Initial BW (g)	855.67	877.63	869.00	40.83	0.930
Final BW (g)	826.93	801.17	809.20	37.19	0.883
Weight loss (g)	28.73	59.80	76.47	15.92	0.133
Pair of breeding pigeon and squabs					
ADFI (g/day)	153.28^a^	148.62^b^	147.31^b^	1.01	0.003
NFCR (g/g)	6.53^a^	5.65^b^	5.94^b^	0.18	0.006
Carcass Characteristics (%)					
Dressing	65.41	65.16	64.53	1.70	0.894
Liver	2.12	2.15	2.32	0.22	0.769
Heart	0.82	0.83	0.802	0.06	0.946
Gizzard	1.82	1.82	1.60	0.12	0.416
Procentrioles	0.24	0.22	0.18	0.04	0.243
Pancreas	0.242	0.342	0.30	0.06	0.558
Bursa	0.18	0.19	0.11	0.03	0.353
Thymus	0.54	0.54	0.48	0.13	0.937
Spleen	0.05	0.05	0.07	0.01	0.088
Abdominal fat	0.52	0.53	0.71	0.05	0.101

*Treatment; T1 = control group; T2 = HM 1%; T3 = HM 2%; HM = Black seeds, Dill, Sage, and Coriander at 1:1:1:1.

Means with different superscripts in the same row differ significantly (*P* < 0.05).

Compared to the control group, breeding pigeons and squabs showed significantly lower average daily feed intake and NFCR with HM (*P* < 0.05). The best NFCR was observed in birds fed 1% HM-supplemented diets.

The birds fed 1% supplemented diets exhibited numerically higher final BW and ADG, and a lower NFCR, than those fed 2% supplemented diets. Furthermore, no significant differences were noted among the HM treatment groups.

The dressing percentage and the relative weights of the liver, heart, gizzard, proventriculus, pancreas, bursa, thymus, spleen, and abdominal fat showed no significant differences (*P* > 0.05) among the various experimental groups.

### Behavioral satiety sequence

**Fig. 1:**
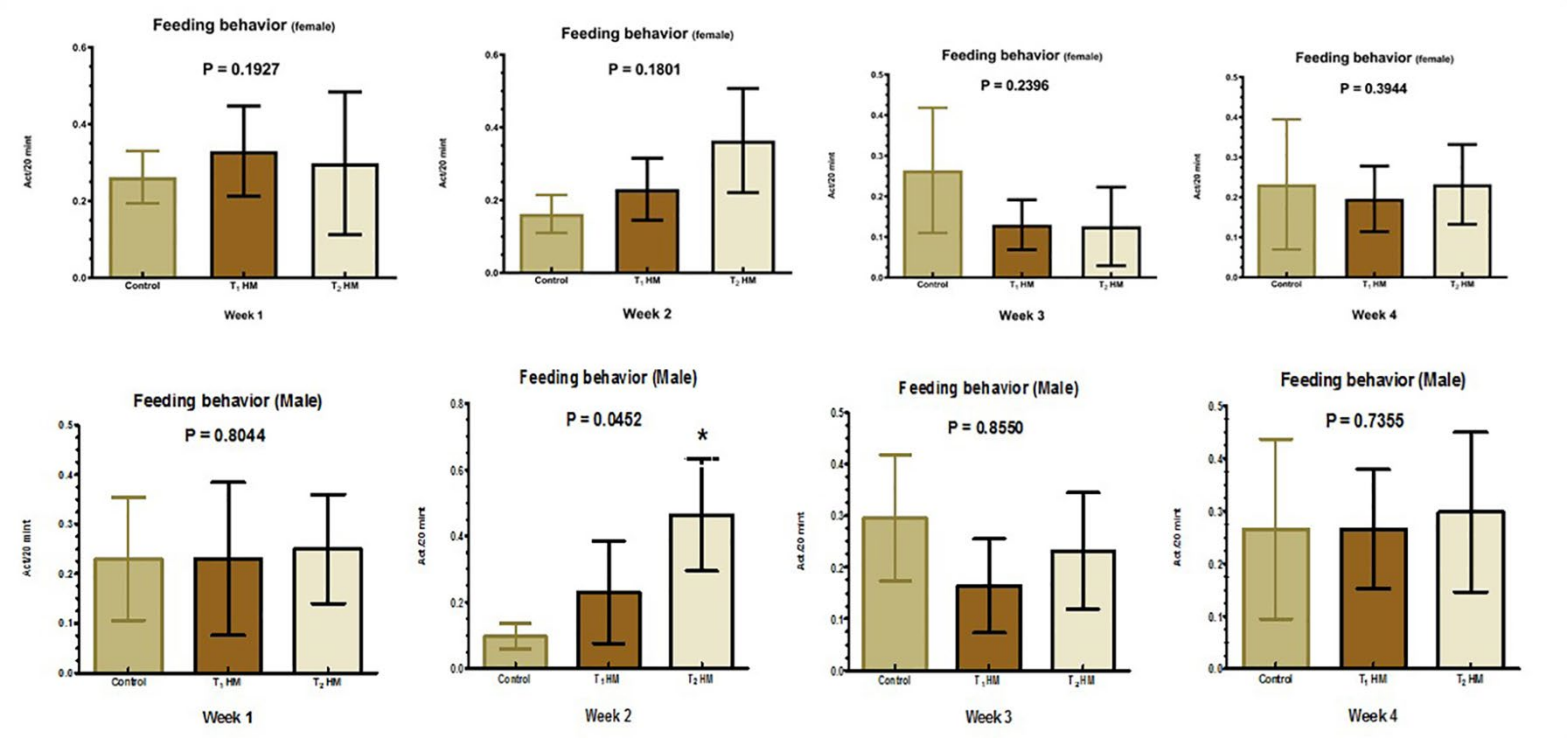
Effect of a HM of black seeds, dill, sage and coriander on the feeding behavior of adult pigeons.

**Fig. 2:**
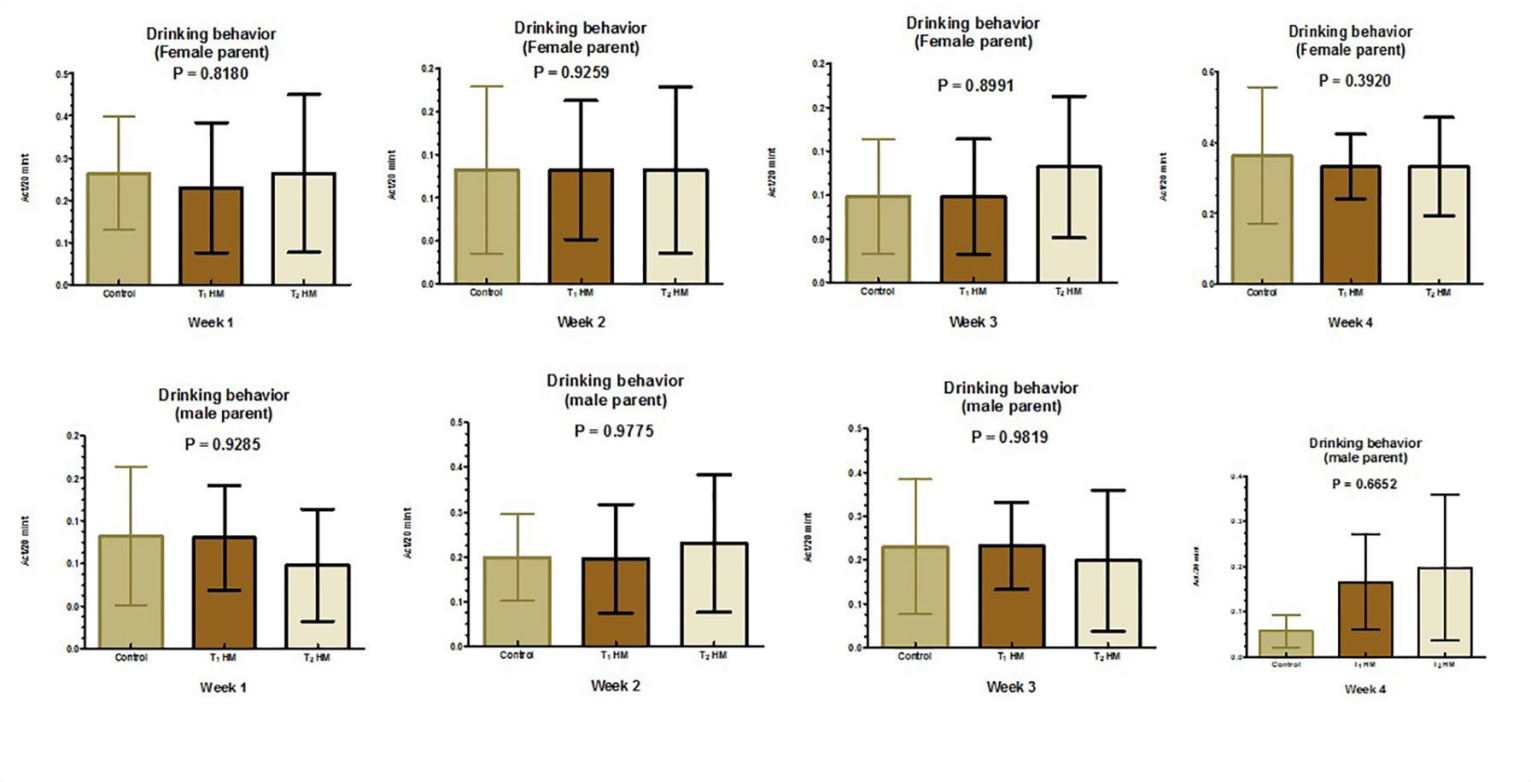
Effect of an HM of black seeds, dill, sage and coriander on the drinking behavior of adult pigeons.

**Fig. 3:**
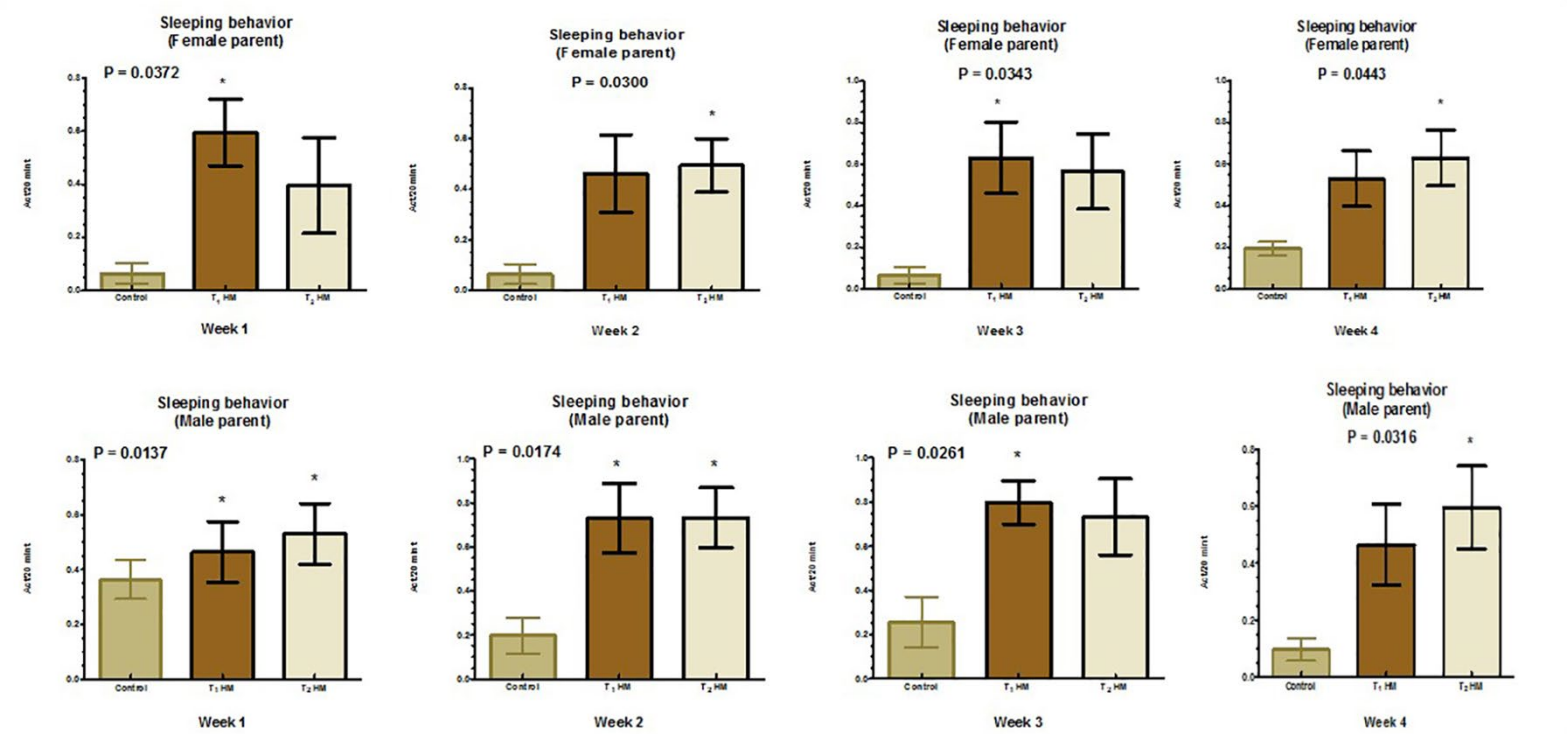
Effect of an HM of black seeds, dill, sage and coriander on the sleeping behavior of adult pigeons.

**Fig. 4:**
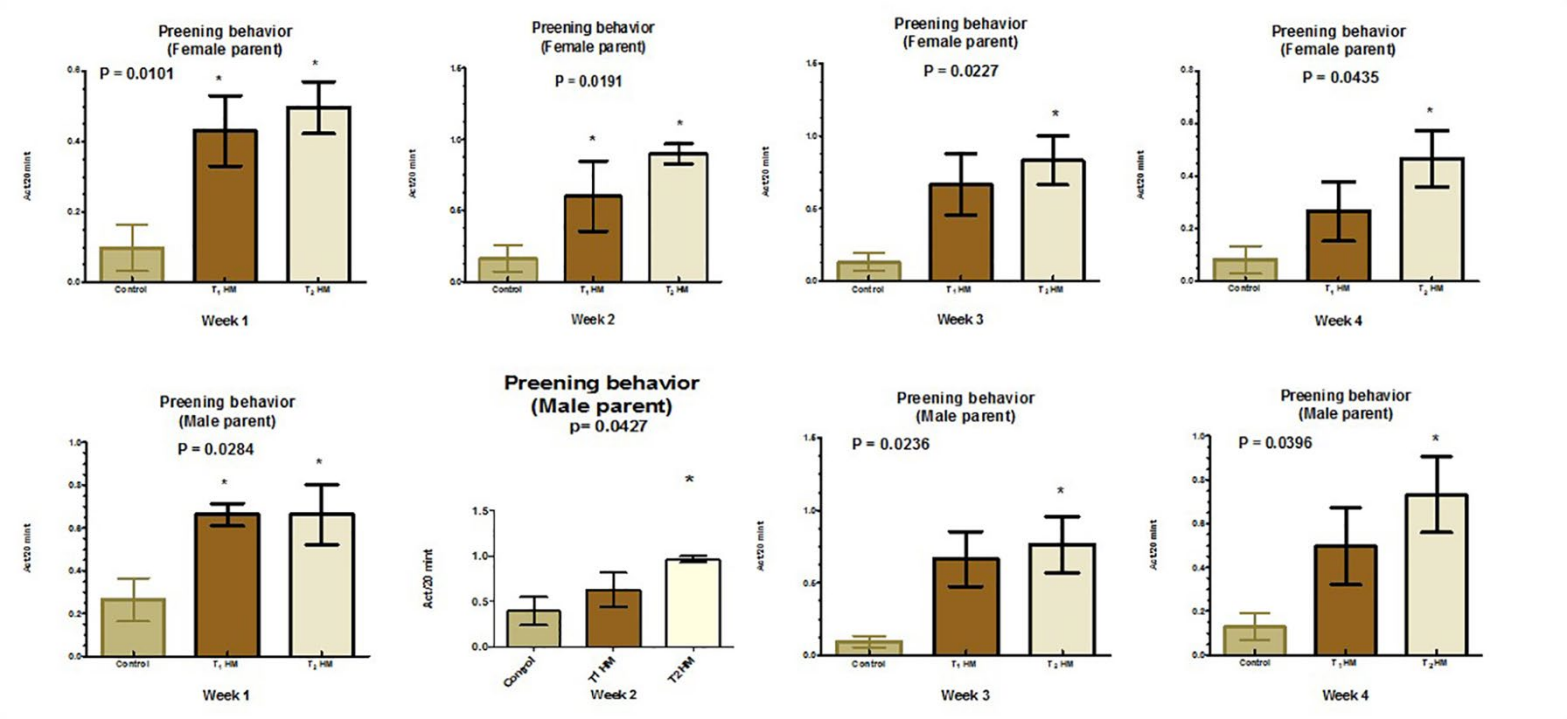
Effect of an HM of black seeds, dill, sage and coriander on preening behavior of adult pigeons.

Figures 1, 2, 3 and 4 illustrate the impact of the HM on the behavioral satiety sequence in parent pigeons. Overall, herbal supplementation did not affect parent pigeons’ feeding and drinking behaviors; however, male pigeons receiving 2% HM demonstrated increased feeding behavior (*P* < 0.05) during the second week. Additionally, both male and female parent pigeons showed improved preening and sleep behaviors (*P* < 0.05) following herbal treatment.

### Serum biochemical indices, antioxidant biomarkers, and Immunoglobulins

The serum biochemical indices, antioxidant biomarkers, and immunoglobulins of pigeon squabs affected by HM are presented in Table 3. Herbal treatments significantly reduced serum T. cholesterol, LDL-C, and triglycerides (*P* < 0.05), while increasing HDL-C levels. Serum enzymes like SOD and GSH-Px showed significant increases, whereas IgG and IgM remained unaffected by the addition of HM.

**Table 3:** Effect of herbal mixture on serum biochemical indices, antioxidant biomarkers, and Immunoglobulins of pigeon squabs.

Items	Treatment*				
	T1	T2	T3	SEM	*P*-value
Total protein (g/dL)	3.41	3.76	3.67	0.17	0.384
Albumin (g/dL)	1.23	1.26	1.18	0.05	0.629
Globulin (g/dL)	2.18	2.50	2.49	0.19	0.448
A/G ratio	0.56	0.51	0.49	0.05	0.609
Triglycerides (mg/dl)	183.33^a^	146.61^b^	154.00^b^	4.04	0.02
T. cholesterol (mg/dl)	377.59^a^	308.27^b^	317.33^b^	7.79	0.001
HDL-C (mg/dl)	30.90^c^	35.50^b^	38.50^a^	0.60	< 0.001
LDL-C (mg/dl)	310.02^a^	243.44^b^	248.03^b^	7.62	0.001
SOD (U/ml)	42.05^b^	60.50^a^	60.00^a^	3.86	0.024
GSH-Px (U/ml)	358.83^b^	410.25^a^	413.70^a^	10.97	0.021
IgG mg/dL	205.91	345.33	363.41	67.85	0.275
IgM mg/dL	11.78	19.60	20.76	3.88	0.281

*Treatment; T1 = control group; T2 = HM 1%; T3 = HM 2%; HM = Black seeds, Dill, Sage, and Coriander at 1:1:1:1.

Means with different superscripts in the same row differ significantly (*P* < 0.05).

A/G ratio; albumin/globulin ratio.

### Meat quality parameters

Table 4 summarizes the effect of HM on meat quality variables. HM supplementation did not affect meat quality parameters, such as pH, color, odor, drip loss, cooking loss, and WHC.

**Table 4:** Effect of herbal mixture on the meat quality of pigeon squabs.

Items	Treatment*				
	T1	T2	T3	SEM	*P*-value
Basic meat quality parameters					
pH	6.02	5.94	5.94	0.04	0.309
Color	7.03	7.09	7.27	0.07	0.164
Odor	7.03	7.02	7.02	0.01	0.842
Drip loss, %	7.43	7.40	7.63	0.08	0.160
Cooking loss, %	22.00	24.67	28.00	1.84	0.147
WHC, %	98.33	97.67	97.00	0.43	0.171
Nutritional meat composition, %					
Moisture	73.74^a^	72.07^b^	72.01^b^	0.24	0.004
Crude protein	20.89^a^	19.14^b^	19.64^ab^	0.39	0.047
Ash	1.27^b^	1.93^a^	1.76^a^	0.06	< 0.001
Fat	2.36^b^	6.70^a^	6.35^a^	0.18	< 0.001
Meat oxidation parameters					
Peroxide value, meq/kg	0.59	0.58	0.62	0.04	0.846
TBA, mg MDA/kg	0.14	0.13	0.14	0.02	0.885

*Treatment; T1 = control group; T2 = HM 1%; T3 = HM 2%; HM = Black seeds, Dill, Sage, and Coriander at 1:1:1:1.

Means with different superscripts in the same row differ significantly (*P* < 0.05).

TBA; Thio-barbituric acid.

HM supplementation altered the nutritional composition of meat, resulting in significant decreases in moisture and crude protein content. In contrast, the percentage of ash and fat increased compared to the control group.

Oxidation analysis includes peroxide value, and TBARS were not affected by HM supplementation.

The composition of amino acids (essential and non-essential) in meat, as influenced by HM, is presented in Table 5. The essential amino acids—valine, methionine, isoleucine, and leucine—as well as the non-essential amino acids, including glutamine, arginine, glycine, cystine, tyrosine, histidine, and proline, were not altered by HM. Herbal treatments significantly reduced lysine and asparagine levels while increasing serine levels. Phenylalanine and alanine decreased, whereas threonine increased with 1% herbal supplementation.

**Table 5:** Effect of herbal mixture on the essential and non-essential amino acids in the meat of pigeon squabs.

Items	Treatment*				
	T1	T2	T3	SEM	*P*-value
Essential amino acids					
Lysine	2.17^a^	2.11^b^	2.11^b^	0.01	0.008
Valine	2.67	2.69	2.64	0.02	0.273
Methionine	0.72	0.75	0.72	0.02	0.433
Isoleucine	2.58	2.52	2.54	0.02	0.120
Leucine	3.02	3.03	3.00	0.01	0.150
Phenylalanine	2.81^a^	2.68^b^	2.83^a^	0.01	< 0.001
Threonine	2.23^b^	2.30^a^	2.24^b^	0.01	0.010
Non-essential amino acids					
Asparagine	3.51^a^	3.21^c^	3.35^b^	0.02	< 0.001
Glutamine	5.89	5.98	5.90	0.04	0.373
Arginine	1.13	1.12	1.12	0.01	0.680
Glycine	2.28	2.28	2.26	0.01	0.332
Alanine	2.81^a^	2.70^b^	2.79^a^	0.01	0.003
Serine	1.79^b^	1.88^a^	1.85^a^	0.02	0.021
Cystine	0.17	0.15	0.14	0.01	0.245
Tyrosine	1.68	1.70	1.65	0.01	0.078
Histidine	0.82	0.83	0.82	0.01	0.880
Proline	0.67	0.65	0.67	0.01	0.469

*Treatment; T1 = control group; T2 = HM 1%; T3 = HM 2%; HM = Black seeds, Dill, Sage, and Coriander at 1:1:1:1.

Means with different superscripts in the same row differ significantly (*P* < 0.05).

### Histomorphometrical data

**Fig. 5:**
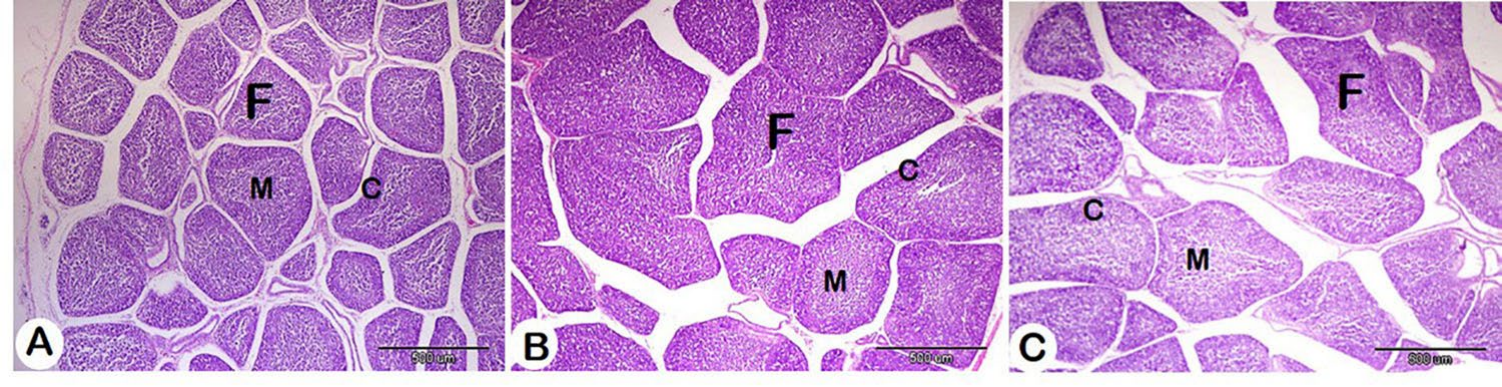
Histomorphometry of the effect of different concentrations of HM on the Bursa. A: Control group, B: HM group 1, and C: HM group 2. Follicle (F), cortex (C), and medulla (M). H&E stain.

**Fig. 6:**
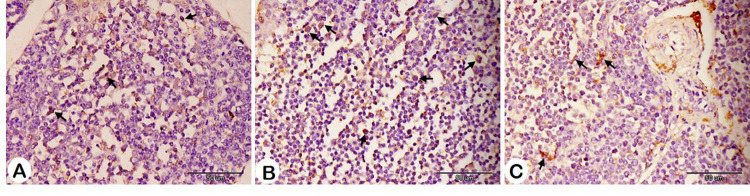
Immunohistochemistry analysis of the bursa showing S100-positive cells (arrows) in different groups. A: Control group, B: HM group 1, and C: HM group 2.

Bursal and spleen histomorphometrical data affected by HM are presented in Table 6. Regarding the bursa, both the area of the follicle and the area of the follicle medulla increased with HM supplementation, and this increase was significant with 1% HM. The control group exhibited the most numerous follicles, followed by the 2% HM group. On the other hand, the most numerous S100-positive cells were detected in the HM groups, with significance in the 1% HM group (Figs. 5 and 6; Table 6).

**Table 6:** Effect of herbal mixture on the bursa and spleen histomorphometry of pigeon squabs.

Items	Treatment*				
	T1	T2	T3	SEM	*P*-value
Bursa histomorphometry					
Area of follicle /um^2^	175801.50^b^	320382.85^a^	225700.27^b^	19131.39	0.005
Area of follicle medulla/um^2^	85138.37^b^	176288.24^a^	99456.23^b^	11648.09	0.003
Area of follicle cortex/um^2^	91881.52	144094.61	112259.95	15868.77	0.142
Number of follicles/500um	25.50^a^	15.83^c^	19.83^b^	0.49	< 0.001
Number of S100 + cells	13.67^b^	25.67^a^	18.67^b^	1.72	0.008
Spleen histomorphometry					
Area of white pulp/um^2^	12688.60^c^	17312.45^b^	38652.48^a^	1105.32	< 0.001
Number of S100 + cells	8.50^b^	7.00^b^	16.33^a^	0.69	< 0.001

*Treatment; T1 = control group; T2 = HM 1%; T3 = HM 2%; HM = Black seeds, Dill, Sage, and Coriander at 1:1:1:1.

Means with different superscripts in the same row differ significantly (*P* < 0.05).

**Fig. 7:**
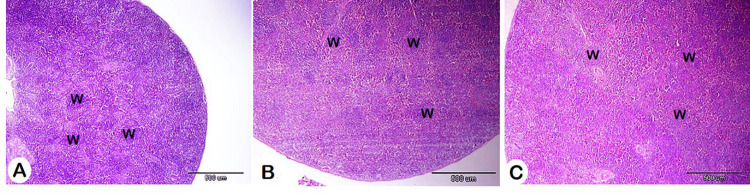
Histomorphometry of the effect of different concentrations of HM on the spleen. A: Control group, B: HM group 1, and C: HM group 2. White pulp (W). H&E stain.

**Fig. 8:**
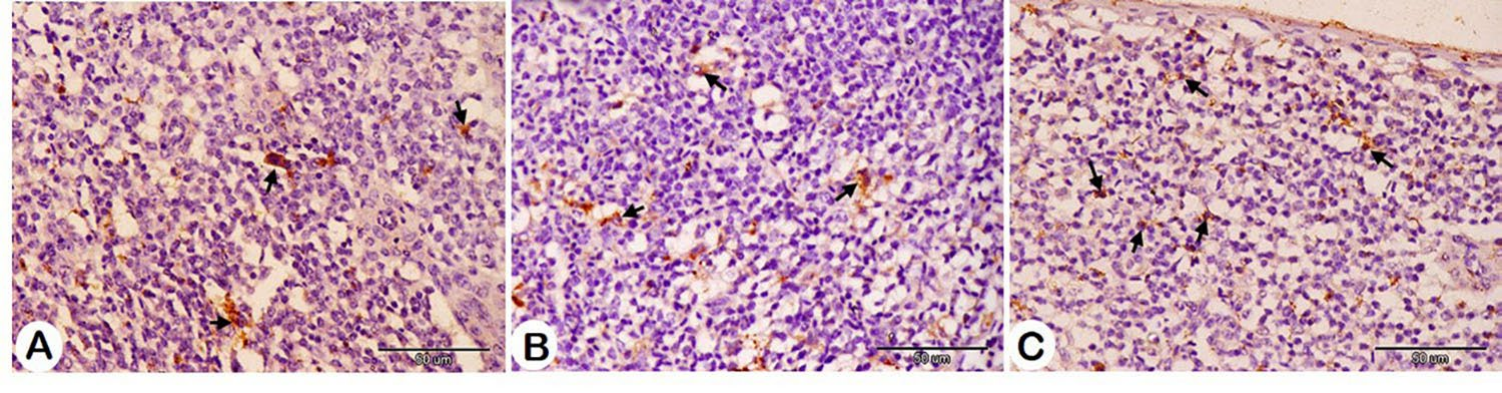
Immunohistochemistry analysis of the spleen showing S100-positive cells (arrows) in different groups. A: Control group, B: HM group 1, and C: HM group 2.

For spleen histomorphometry, the results showed a significant increase in the white pulp area with HM. Additionally, the number of S100-positive cells was significantly higher in the 2% HM group compared to both the control and 1% HM groups (Figs. 7 and 8; Table 6).

### Economic efficiency

Table 7 provides a comparative economic evaluation of the experimental diets, detailing feed costs and returns. Compared to the control group, birds fed 1% HM showed an increase in net revenue, along with improvements in EE and relative EE, followed by those fed 2% HM.

**Table 7:** Effect of herbal mixture on the economic evaluation of pigeons.

Items	Treatment*		
	T1	T2	T3
Feed cost/bird, EGP	35.41	34.67	34.71
total revenue, EGP	238.83	245.47	245.20
Net revenue, EGP	33.42	40.80	40.49
EE	16.27	19.93	19.78
Relative EE	100	122.52	121.57

*Treatment; T1 = control group; T2 = HM 1%; T3 = HM 2%; HM = Black seeds, Dill, Sage, and Coriander at 1:1:1:1.EGP; Egyptian Pounds. EE; Economic Efficiency.

## Discussion

Supplementing animal diets with feed additives, such as herbs, can increase feed shelf life, improve animal health, and enhance the quality of products such as meat, milk, wool, and eggs^54^. Herbs, rich in bioactive compounds such as phenols, polysaccharides, and essential oils, and their various effects, including antioxidant, immunomodulatory, anti-inflammatory, and antimicrobial, have attracted the attention of many scientists in recent years^55^.

In our trial, HM had a positive effect on growth performance parameters. The BW and ADG of pigeon squabs increased, average daily feed intake decreased, and NFCR improved. Our results were in agreement with those of Abd El-Hack et al.^56^, El-Kashef^57^, Asghar et al.^13^, Yassein et al.^58^, Abdullah et al.^59^, AL-Hayani et al.^60^, and Ashour et al.^61^ reported an enhancement in birds’ growth performance with herbal supplementation and suggested that this improvement was due to increased nutrient digestibility and absorption resulting from the herbs. Herbs positively influence digestion through their essential oils and support the activity of intestinal and pancreatic enzymes^62^.

Increased weight gain and improved feed conversion ratios (FCR) were observed in broilers fed black seeds^11^, dill^63^, sage^64,65^, and coriander^66,67^.

It was identified that the herbal bioactive substances responsible for growth-promoting effects include quercetin, kaempferol, and ellagic acid in black seeds^12^, carvone and limonene in dill^68^, carotene, tannins, alkaloids, and saponins in sage plants^22^, and linalool compounds in coriander^67^. All these bioactive compounds act as appetite stimulants, inhibitors of gastrointestinal microbes, and enhancers of nutrient absorption, thus promoting animal growth and overall health^12,22,67,68^. Moreover, the bioactive compounds in dill, flavonoids, can play an essential role in preventing tissue degradation and enhancing protein synthesis^69^.

However, supplementing broilers’ diets with black seeds^70^, dill^15^, sage^21^, and coriander^71,72^ in different studies did not impact growth performance parameters.

Regarding the traits of the squab’s carcass, HM did not affect dressing percentage, abdominal fat, or internal organ weight. Our results were aligned with several studies indicating that adding black seed^73^, dill^74^, sage^75^, or coriander^67,72^ to a bird^’^s diet did not influence carcass traits. Conversely, internal organs and carcass traits in other studies were positively affected by coriander supplementation^76^ and sage powder^77^.The variability of herbal effects across poultry trials may stem from differences in extraction methods, dosage levels, environmental conditions, or genetic differences among bird breeds.

Behavioral data indicated that HM did not affect the feeding and drinking of parent pigeons, except in 2% of HM, where male pigeons showed increased feeding during the second week. The increase in feeding behavior in males may be due to HM enhancing digestion, nutrient digestibility, and gut health [20, 21, 26–28]. Moreover, it’s possible that male pigeons exhibit better results than females since they are endued with more homo-fermentative lactic acid bacteria. Male animals’ guts contain a more beneficial microbiota, as evidenced by the proximal and distal ileum’s concurrently decreased goblet cell counts and the distal ileum’s increased villus surface area^78^. Multiple studies have found that herbs do not significantly impact feeding and drinking behavior, including black seed^79^, dill seed^15^, sage powder^21^, and coriander seed^80^.

Regarding preening and sleeping behaviors, HM improved these activities in birds. The calming and hypnotic effects of linalool oil, which improve sleep latency and lessen sleep disturbances^81^, as well as its anti-anxiety qualities^82^, may be the reason for the increase in sleeping and preening behavior in the case of HM addition. In addition, by regulating sleep/wake cycles, controlling cortisol/melatonin balance through Hypothalamic–Pituitary–Adrenal axis regulation, and activating Gamma-aminobutyric acid in the central nervous system, black cumin oil (BCO-5) reduced stress and enhanced the quality of sleep^83^. By raising the levels of 5-hydroxy tryptophan and tryptophan in the brain, BCO-5 can raise melatonin levels. This, in turn, raises serotonin levels, the biosynthetic precursor of melatonin^84^. These data were aligned with those of Lima Giacobbo et al.^85^ and Mima et al.^86^, detected improvements in these behaviors and overall health with herbs. In contrast to these findings, EL Shoukary et al.^79^, who added black seed to the diet of pigeons, did not record any significant herbal effects on these self-maintaining behaviors. This may suggest that bird behaviors regarding the addition of herbs to their diets vary depending on the herb itself.

Birds’ health and metabolism are indicated by levels of total proteins, albumin, and globulin in their blood^87^. In our experiment, blood proteins were not affected by HM. Consistent with these findings, black seed^88,89^, hemp and dill seeds^15^, sage powder^77^, and coriander seed^72^ did not impact these parameters when added to poultry diets. The lack of a significant effect on blood protein concentrations may be explained by the fact that HM does not disrupt protein metabolism or hepatic synthetic activity. Additionally, the bioactive components of these additives, which include phenolic compounds, flavonoids, terpenoids, and essential fatty acids, mainly function as antioxidants and anti-inflammatory agents^90^. Therefore, the stable blood protein profile observed in this study due to HM inclusion reflects normal liver function and balanced protein metabolism.

Herbal supplementation had an adverse effect on T. cholesterol, LDL-C, and triglycerides, and a positive effect on HDL-C levels. Al-Jaff^91^ and Farhadi et al.^65^ reported similar findings with the supplementation of coriander and sage powder. The herbal ability to lower cholesterol and triglyceride levels in blood is linked to their bioactive substances, which can inhibit the absorption of cholesterol and unsaturated fatty acids, promote their excretion in the intestine as with black seeds^57,92^, or block hepatic cholesterogenic enzymes such as cholesterol-7α-hydroxylase as with dill seeds^15^, or help regulate lipid metabolism^93^.

Herbs can affect antioxidant activity by increasing the expression of genes that encode antioxidant enzymes^93^. In our study, herbal supplementation increased the level of the antioxidant indicators SOD and GSH-Px. These enzymes are characterized by their exceptional ability to detoxify free radicals^94^. Various studies have reported that these enzymes can be stimulated by several herbs: black seeds^11^, dill^94^, sage extract^95^, and coriander^96^. The antioxidative effects of herbs in birds, including the activation of enzymes, inhibition of lipid peroxidation, and scavenging of superoxide anions, are attributed to their bioactive components, such as ascorbic acid, flavonoids, and polyphenols^96,97^.

Regarding immunity and immunoglobulin response to herbal supplementation in our study, IgG and IgM showed non-significant numerical increases. Our results were not consistent with previous studies that reported a positive effect of herbs on bird immunity, such as black seed^98^, coriander^99^, and dill^21^. The variation in results between our study and others may relate to the type of herbs used, the level of herbs, the species fed the herbs, and the experimental design used.

Bird immunity can be boosted by herbs through various direct and indirect mechanisms. The herbal bioactive compounds, such as flavonoids and terpenoids, can directly influence immunity^96^ by stimulating immune cells^93^, indirectly by increasing immunoglobulin production, or by reducing oxidative stress^7,11^. Furthermore, Jiang et al.^100^ noted that herbal compounds can improve gut microbiota by suppressing harmful bacteria and promoting beneficial bacteria. This enhances digestion and absorption, which, in turn, helps modulate immunity and the immune response.

Generally, the meat quality parameters in our study were unaffected by HM, showing normal levels similar to those of the control. The meat pH values in the control and HM groups ranged from 5.94 to 6.2. Fu et al.^101^ noted that birds fed herbs had lower stress and less glycogen depletion before slaughter, which helped maintain meat pH within the normal range (5.7-6.0). Similarly, the values for color, odor, drip loss, cooking loss, WHC, peroxide value, and TBARS were similar to those of the control and were not affected by HM.

In their herbal studies^38,101–105^, researchers found that herbs can positively influence meat quality by delaying oxidation, reducing lactic acid buildup, and preventing volatile compound formation, thereby helping protect meat proteins.

The chemical makeup of poultry meat can be improved through herbal supplementation, affecting its protein, fat, fatty acids, amino acids, and minerals^102^. Our study showed that fat and ash levels increased, moisture and crude protein levels decreased, and the composition of essential and non-essential amino acids was altered with HM supplementation. Bonos et al.^106^ observed an increase in meat fat content when sage and oregano were added to broiler diets. They suggested that bioactive substances in herbs play a crucial role in preserving broiler meat and extending its shelf life by regulating the meat’s lipid profile through the inhibition of Polyunsaturated Fatty Acids oxidation and the reduction of saturated fat. Although meat protein content decreased in our experiment, Liu et al.^107^ reported that herbs can positively affect meat protein by stimulating genes involved in protein metabolism in birds.

Histomorphometrical results demonstrated significant effects of dietary HM supplementation on numerous parameters in the bursa. The area of the follicle in both HM groups was increased with HM supplementation, with a particularly significant enlargement detected in the 1% HM. This indicated that HM may promote follicular expansion, potentially enhancing immune cell activity and responses, as the Bursa of Fabricius is a unique constituent of the bird immune system and plays an important role in B lymphocyte development^108,109^. Also, the area of the follicle medulla was enhanced in the HM groups and increased significantly in the 1% HM compared to the other groups. This indicates that dietary supplementation with HM, especially at lower doses, can support the medullary region’s development. Previous studies support our findings. For example, black seed supplementation increased the size of bursal follicles and enhanced the immunity of broiler chickens^110^. Similarly, sage powder increased the weight of the Bursa of Fabercius^111^. In addition, coriander seed powder supplementation increased the bursa weight of broilers and is suggested to have immune stimulant effects^67^.

The spleen is one of the most important immune organs, plays a vital role in the immune response, and is considered the largest lymphoid tissue^112,113^. Spleen tissue analysis following HM supplementation indicated dose-dependent variations in histomorphometrical data. The white pulp area in both HM groups increased significantly compared to the control, with the 2% HM showing the most marked enlargement. This indicated that HM at higher doses may influence white pulp area architecture, possibly improving immune function, as the white pulp is lymphoid tissue responsible for several immune functions, such as containing immune cells that target blood-borne pathogens^114^. Our findings were supported by earlier research. For instance, using of black seeds in the diet enhances immunity and increases the size of the spleen and bursa in broiler chickens^115^. Similarly, black seed supplementation in the diets of rat increases the area of the splenic white pulp^116^. In addition, sage powder induced immune cells and spleen weight in rabbits^117^. Also, in broiler chickens coriander seed supplementation had a pronounced outcome on the spleen weight of the^118^.

Black seeds, dill, sage, and coriander are well-known to have immunomodulatory effects. Black seeds have an immunomodulatory role through the induction of cellular immunity by inducing T lymphocyte proliferation and increasing total leukocyte count^119^. Furthermore, it promotes the synthesis of interferon and stimulates bone marrow cells^116,120^. In addition, dill supplementation has been reported to increase white blood cell numbers^121^. Sage enhanced humoral and cell-mediated immune response^122^. Coriander has been shown to improve innate immunity^123^.

The S100-positive cell counts in the bursa were higher in both HM groups, with 1% of HM showing a significant increase. Additionally, 2% HM demonstrated a significant rise in S100-positive cells in the spleen compared to the control and 1% HM. This suggests an enhancement of immune cell activity in the bursa and spleen following HM supplementation. The S100 protein, a low-molecular-weight calcium-binding protein, plays crucial roles in inflammation, immune responses, and calcium homeostasis. It acts as an antimicrobial agent, a chemoattractant, and a pro-inflammatory stimulator^124^. S100 was detected in birds at different ages in prior studies and is found in follicular dendritic cells in the spleen and eosinophilic granulocytes, dendritic cells, and reticular cells of the bursa^125–127^.

The economic assessment demonstrated that HM, particularly at the 1% level, was more cost-effective than the control diet. This improvement may result from reduced feed required to produce one unit of meat or enhanced feed conversion efficiency. These findings aligned with previous research showing that herbal supplementation improved the economic values of bird diets^64,69,128^.

Despite this study providing novel results for the impact of adding HM in pigeon nutrition, some limitations should be acknowledged. The available literature on herbals and HMs in pigeon nutrition is rare, which makes direct comparison difficult. The available literature on histological effects on immune organs is scarce in birds, and almost nonexistent in pigeons. Future studies with longer experimental periods and more sample size at different ages of pigeons and under different seasonal and physiological conditions are needed. Moreover, insertion of various histological, immunohistochemical, and molecular procedures is important to support the data and explain in depth the biological pathways in feeding experiments.

## Conclusion

Incorporating 1% HM positively influenced pigeon parents’ behavior, squab performance, and the economic return. It also improved blood metabolites, antioxidant status, immunity, meat quality, and bursal and spleen architecture. Therefore, 1% HM may be considered a practical and sustainable strategy to enhance productivity and immunity in pigeon production.

## Data Availability

The datasets utilized and/or examined in the present investigation can be obtained from the corresponding author upon a reasonable request.
